# Insights from the global education survey on the use of VR-haptics in dental education

**DOI:** 10.3389/fdmed.2025.1576646

**Published:** 2025-04-24

**Authors:** Sompop Bencharit, Barry Quinn, Maria F. Sittoni-Pino, Santiago Arias-Herrera, Simona-Georgiana Schick, Sarah Rampf, Samantha Byrne, Muhammad A. Shazib, Ulf Örtengren, Walter Yu Hang Lam, Mikko Liukkonen, David Rice, Masako Nagasawa, Amitha Ranauta, Sobia Zafar, Kinga Bágyi, Thomas J. Greany, Amirul Faiz Luai, Marit Øilo, Gitana Rederiene, Rebecca Stolberg, Gülsün Gül, Jorge Tricio, Reinhard Chun Wang Chau, Mihaela Pantea, Murat Mutluay, Peter Lingström, Ophir Klein, Sıla Nur Usta, Liisa Suominen, Szabolcs Felszeghy

**Affiliations:** ^1^ Workman School of Dental Medicine, High Point University, High Point, NC, United States; ^2^ School of Dentistry, University of Liverpool, Liverpool, United Kingdom; ^3^ Faculty of Health Sciences, Department of Dentistry, Universidad Europea de Valencia, Valencia, Spain; ^4^ Clinic for Oral, Dental and Maxillofacial Diseases, Department of Conservative Dentistry, Heidelberg University, Heidelberg, Germany; ^5^ Melbourne Dental School, University of Melbourne, Melbourne, VIC, Australia; ^6^ Department of Cariology, Institute of Odontology, Sahlgrenska Academy, University of Gothenburg, Gothenburg, Sweden; ^7^ Prosthodontics, Faculty of Dentistry, The University of Hong Kong, Hong Kong, Hong Kong SAR, China; ^8^ Institute of Clinical Medicine, School of Medicine, University of Eastern Finland, Kuopio, Finland; ^9^ Department of Oral and Maxillofacial Diseases, University of Helsinki and Helsinki University Hospital, Helsinki, Finland; ^10^ Faculty of Dentistry & Graduate School of Medical and Dental Sciences, Division of Bio-Prosthodontics, Niigata University, Niigata, Japan; ^11^ Queen Mary University of London, London, United Kingdom; ^12^ School of Dentistry, The University of Queensland, Brisbane, QLD, Australia; ^13^ Faculty of Dentistry, University of Debrecen, Debrecen, Hungary; ^14^ School of Dental Medicine, University of Colorado Anschutz Medical Campus, Aurora, CO, United States; ^15^ Centre of Population Oral Health and Clinical Prevention Studies, Faculty of Dentistry, Universiti Teknologi MARA (UiTM) Sungai Buloh, Selangor, Malaysia; ^16^ Dental Public Health Unit, Department of Family Oral Health, Faculty of Dentistry, Universiti Kebangsaan Malaysia (UKM), Kuala Lumpur, Malaysia; ^17^ Department of Clinical Dentistry, University of Bergen, Bergen, Norway; ^18^ European Dental Hygienists Federation, Utrecht, Netherlands; ^19^ American Dental Education Association, Washington, DC, United States; ^20^ Faculty of Dentistry, Universidad de los Andes, Santiago, Chile; ^21^ Faculty of Dentistry, “Carol Davila” University of Medicine and Pharmacy, Bucharest, Romania; ^22^ Institute of Dentistry, School of Medicine, University of Eastern Finland, Kuopio, Finland; ^23^ Program in Craniofacial Biology, Department of Orofacial Sciences, University of California, San Fransisco, CA, United States; ^24^ Department of Pediatrics, Cedars-Sinai Guerin Children’s, Los Angeles, CA, United States; ^25^ Department of Endodontics, Gulhane Faculty of Dentistry, University of Health Sciences, Ankara, Türkiye; ^26^ Oral Health Teaching Unit, Kuopio University Hospital, Kuopio, Finland

**Keywords:** dental education, challenges, haptic technology, implementation barriers, virtual reality

## Abstract

**Background:**

Haptics-enhanced virtual reality (VR-haptics), a supplementary tool for traditional oral health training, shows promise in enhancing knowledge acquisition, manual dexterity, performance, and student well-being.

**Aim:**

The aim of this study was to understand dental educators' perceptions and needs regarding the acceptability and application of VR-haptics in dental education, as well as to gather suggestions for system improvements.

**Methods:**

In this global cross-sectional study, the VR-Haptic Thinkers Consortium used a 28-item online questionnaire distributed to 1,023 participants by August 1, 2024. The survey included questions on general demographics, multiple choice and five-point Likert-style questions, and open-ended questions.

**Results:**

A total of 378 responses were collected from 156 institutions. 57% of respondents had a dental doctorate degree and 59% had a PhD. VR-haptic trainers were used more often in preclinical training (94% of responses) than clinical training (46%). The three most common course types with VR-haptics incorporation were restorative, prosthodontic, and endodontic courses. Most respondents thought that the best approach to implementing VR-haptics is alongside phantom head training in the preclinical stage (58%). A third of the feedback on the challenges in VR-haptics utilization in dental training highlighted a need for further hardware and software development, while more than one-fourth cited economic issues in system acquisition and housing, and another one-fourth reported low acceptance of the technology among educators and students. The most mentioned enhancement requests for dental trainers were more diverse training scenarios (20%), improved software (19%) and hardware (19%) elements, and advancements in AI-based personalized training and monitoring (18%). Additionally, 10% of respondents suggested gamification features.

**Conclusions:**

VR-haptic technology is constantly evolving and will likely become more and more accepted as an integral part of dental hand skill development to complement traditional preclinical training. Future research and development should emphasize transitioning from preclinical to clinical restorative, prosthodontic, endodontic, and implantology procedures as part of individualized education and patient care.

## 1 Introduction

Dental education has never hesitated to adapt itself to innovative technologies. Oral health educators have continuously embraced technological advances to meet evolving student needs, competencies, and professional standards to ensure high-quality education (1). Dental students' learning and hand skill development begins in the preclinical training phase, transitioning to professional roles in later phases. Preclinical training fosters a sense of belonging and skill development essential for motivation and preparation for the clinical years (2, 3).

Traditionally, practical dental education utilizes teaching strategies such as operating on manikins to enable new students to practice dental procedures and enhance the retention of knowledge. Immersive technologies, such as virtual reality (VR) and augmented reality (AR), have the potential to provide interactive, engaging, and immersive experiences that enhance education, prevention, and patient care (4, 5). Basic forms of virtual simulation have existed for many decades thanks to the military and aviation sectors' needs (6). However, a wider adaptation of VR and AR educational methodologies in engineering, medical, and other fields has only become reality in the past two decades thanks to advances in computational and display technologies (7–10). VR technologies with added force feedback methods, VR-haptics, hope to supplement some of the aspects where physical training methods have been perceived to be lacking (11). Haptics-enhanced dental trainers were introduced in the early 2010s, with global interest in VR-haptics having surged since 2020, driven by increased accessibility and acceptance post-COVID-19 (12). A previous consensus-based report elaborated on the benefits of VR-haptic methodology employed to create three-dimensional (3D) virtual replicas of operable structures combined with haptic sensory feedback (13). Studies have examined the effectiveness of VR-haptics and students' perceptions of it in both medical and anatomical education, finding that its use seems to enhance student motivation and engagement compared to traditional methodologies, while improving comprehension (14–16).

In 2024, the Virtual Reality-Haptic Thinkers Consortium (VRHTC) was established (17, 18). The consortium has experienced significant growth, doubling its membership and number of participating institutions within the first year, fueled by the renewed interest in accelerated educational pathways supported by VR-haptic technology. The global consortium includes educators and researchers from 41 dental schools that have integrated traditional dental training with VR-haptics-based simulations in their curricula.

Research indicates that this approach not only enhances motor skill acquisition in dental training but also potentially improves overall learning outcomes and mental well-being (5, 19–23). These advancements could allow students to enter their clinical training years feeling less stressed and better prepared (24–26). Incorporation of VR-haptic technology into dental curriculum enhances students' manual dexterity and knowledge acquisition, yet global acceptance among oral health professionals remains underexplored (25–31). Additionally, understanding teaching styles and traits can guide VR-haptic curriculum development, improving learning outcomes. Addressing these knowledge gaps through targeted surveys and curriculum development can drive industrial and educational advancements, shaping the future of dental training.

In our search for a comprehensive survey of educator's perspectives on VR-haptics in dental education, we were confronted with a lack of such work. Most existing studies have concentrated on the development and testing of VR-haptic applications and their implementation in dental curricula, primarily assessing single-centered participants' viewpoints and the local potential educational impact, thus potentially lacking in global applicability. There is a pressing need for more empirical research that focuses specifically on teachers, gaining an even deeper understanding of their perspectives, attitudes, abilities, and readiness to integrate innovative solutions like VR-haptics. Furthermore, understanding the applications of VR-haptics in various disciplines such as restorative dentistry, prosthodontics, endodontics, and implantology can provide a better foundation for future collaboration and broader applications of the technology. Therefore, we initiated a validated anonymous global cross-sectional online survey to explore and understand oral health educators' perceptions on VR-haptic training in preclinical and clinical dental courses. The aim of this study was to provide an overview of the current landscape of challenges and future innovation needs in VR-haptics, along with their distinctive characteristics.

## 2 Materials and methods

This global validated survey was classified as a non-human subject program evaluation, and it followed the guidelines of the European and Finnish National Board on Research Integrity. Ethical approval was obtained from the University of Eastern Finland Committee on Research Ethics (Approval No. 13/2023).

In early 2024, a 28-item online mixed-method questionnaire was created in English by expert educators who have experience in the use of haptic simulators in dental education. Spanish and Japanese translations were also acquired from native speaker colleagues. It included questions on general demographics, multiple choice and five-point Likert-style questions (47, 48), and open-ended questions regarding oral health educators’ perceptions on VR-haptic training.

The voluntary, anonymous online survey was broadly distributed to the members of VRHTC and through social media (X, Instagram, LinkedIn). Non-core VRHTC respondents were incentivized to join with a draw for three monetary prizes (250€ each) to aid them to participate in the 2025 VR-Haptic Thinkers Meetup in London.

The purpose of the study was to gather information on oral health educators' perceptions of VR-haptic training in various dental preclinical and clinical courses. As such, the survey included questions on such topics as the number of preclinical and clinical courses that implement VR-haptics and the challenges related to the integration and use of VR-haptics in the dental curricula. The survey questions and (where available) answer choices have been included as a Supplementary File S1.

Qualitative data obtained from the open-ended questions underwent thematic analysis. The responses were systematically coded based on the data and organized into overarching themes, which were further refined and categorized into sub-categories as necessary to provide a more detailed understanding of the data.

## 3 Results

### 3.1 Respondent demographics

**Figure 1:**
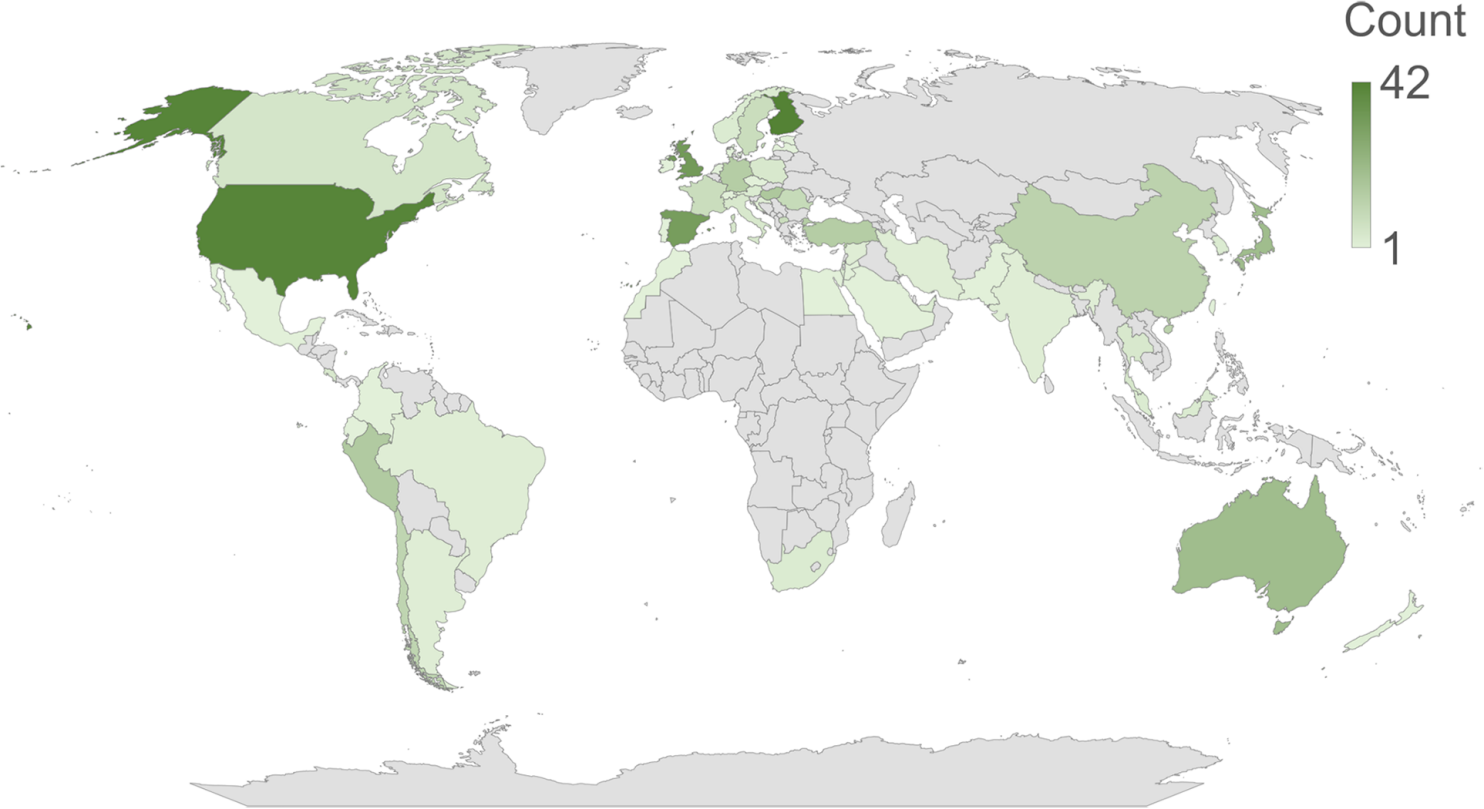
Distribution of the responses based on their country of origin.

**Figure 2:**
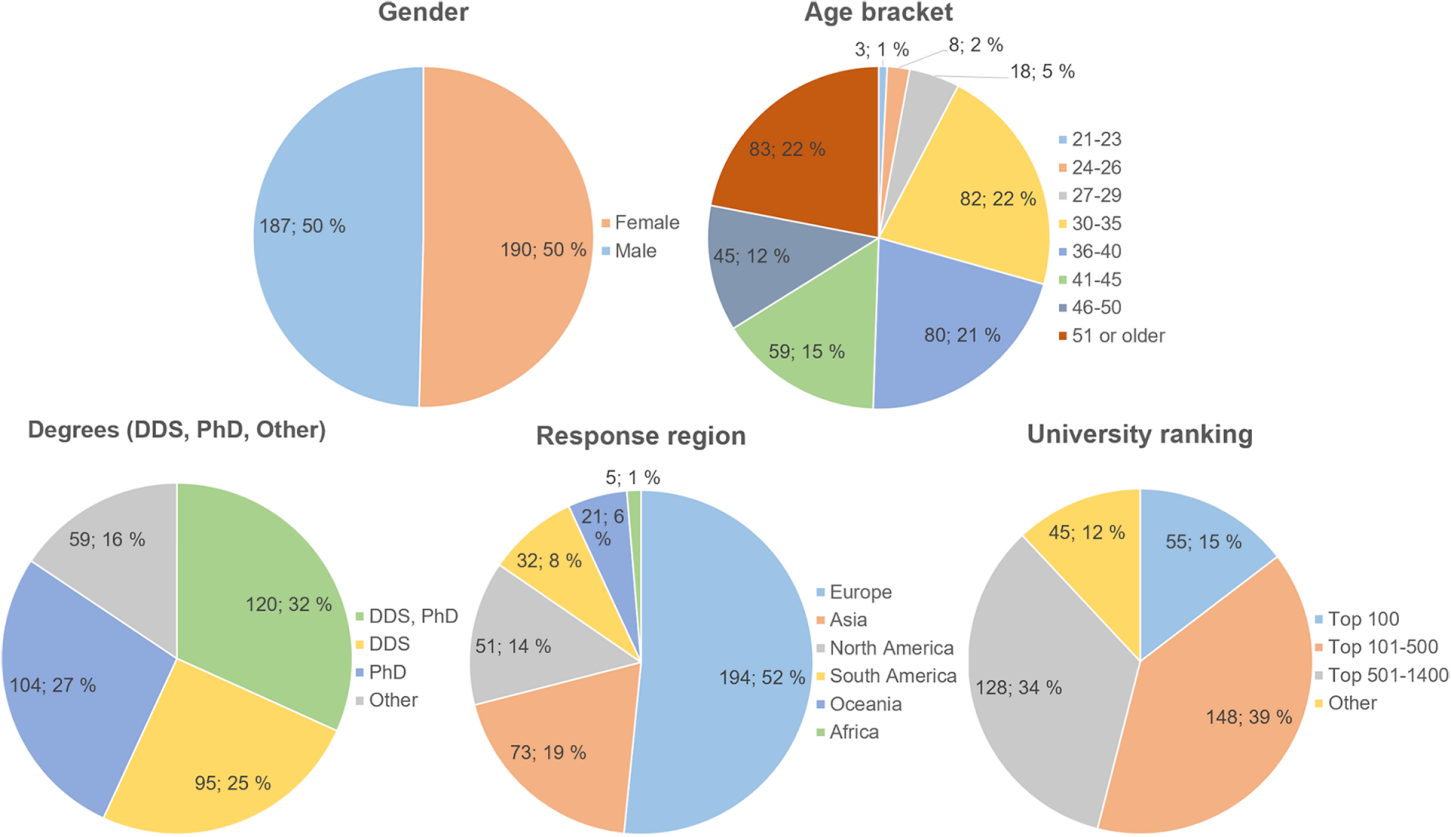
Survey respondent demographics. Gender data omits one “Prefer not to say” response. Scholarly degree data has been simplified to just DDS, PhD, and Other (95% bachelor's and master's) degrees, respondents may have reported other degrees as well. University rankings are based on the QS World University Rankings 2024 data. Data are shown as *n*; %.

A total of 378 responses were collected from 156 institutions across the world (Figure 1). The respondents had an even gender distribution, roughly half of them were aged 41 or over, and 84% had a PhD and/or DDS degree (Figure 2). Of the remaining 16%, 95% consisted of bachelor's and/or master's degree holders, as well as three other education type replies. More than half of the replies were received from universities ranked 500 or higher on the QS World University Rankings 2024.

### 3.2 Use of VR-haptics in dental education

Among the 273 respondents who indicated using VR-haptics in dental education, 37% (100) use it solely as a supportive or adjunct educational tool. Only 4% (11) use it for a specific purpose, with half of them (6) using it as a manual dexterity skills evaluation tool and half (5) as an exclusive training method. The remaining 59% (162) of respondents stated mixed utilization of these applications. 99% (161) of the mixed utilizers incorporated this technology as a supportive tool.

A total of 283 respondents stated that VR-haptic trainers are employed as a supplementary component in dental education. The majority (94%; 266) of respondents indicated their use in preclinical sessions. These tools are commonly utilized in predoctoral (46%; 131) and advanced dental education training including specialist and residency programs (36%; 102). However, their use in interprofessional education remains uncommon (3%; 9).

### 3.3 Integration of VR-haptics in preclinical and clinical training

Of the 373 responses to the question about the types of courses where VR-haptic dental trainers are used in preclinical education, 289 responses identified at least one course or discipline. For clinical education responses, the respective figures were 363 and 245. On average, respondents mentioned 3.2 (SD 1.6, range 1–8) different types of courses for both preclinical and clinical courses. The most cited courses using VR-haptics were restorative dentistry, prosthodontics, and endodontics (Table 1).

**Table 1:** The types of courses incorporating VR-haptics in preclinical and clinical dental education.

Course type	*n* (preclinical/clinical)
Restorative	434 (237/197)
Prosthodontics	384 (200/184)
Endodontics	355 (187/168)
Restorative cariology	193 (110/83)
Implantology	103 (55/48)
Pediatric dentistry	103 (56/47)
Periodontics	96 (54/42)
Other	23 (15/8)

The timing of VR-haptics integration in preclinical dental education is a topic of interest. Of the 372 respondents, 58% (214) thought that VR-haptics should be implemented concurrently with phantom head training to maximize its benefits. Another 30% (111) preferred starting motor skill training with VR-haptics before moving to phantom head simulation. Less than a tenth (9%; 35) thought that it is best to introduce VR-haptics after phantom head training, while only 3% suggested reserving its use for the clinical training phase.

### 3.4 Challenges in implementing VR-haptics

When asked to identify the challenges impeding the implementation of VR-haptics in dental education, a total of 421 responses were received, covering both preclinical (222) and clinical (199) aspects. A total of 436 different challenges were mentioned by the respondents (237 for preclinical and 199 for clinical), which were categorized into 4 different themes: missing or lacking features, high overall cost, lack of acceptance and dismissive perceptions, and the amount of time required for implementation (Table 2). As the main challenge was related to hardware and software needing improvements or additions, it was further divided into 13 sub-categories. The most frequently cited missing features included a lack of clinical metrics and easily accessible performance analysis tools, insufficient realism or precision, and the absence of high and low-speed handpiece options.

**Table 2:** Themes related to the perceived challenges in using VR-haptics in preclinical and clinical dental education.

Challenges	Total *n* (preclinical/clinical)
Missing features or needs improvement (hardware, software)	151 (75/76)
Economics (expense, low unit number, space requirements)	121 (73/48)
Acceptance (other educators, faculty, students) and perceptions	106 (54/52)
Time (adaptation, learning) and curriculum integration (scheduling)	58 (35/23)
Missing features or needs improvement, sub-categories	
Missing clinical metrics and easily accessible performance analysis options	53 (25/28)
Insufficient realism or precision	29 (11/18)
Lack of high and low-speed options	24 (12/12)
Better (AI-powered?) personalization options needed	17 (10/7)
Needs voice feedback and/or voice controls	12 (7/5)
More portable devices required	11 (7/4)
More training scenarios needed	6 (2/4)
Transfer of real-life cases too difficult	6 (0/6)
Missing instruments	4 (1/3)
More gamification options needed	4 (4/0)
Accessibility issues	3 (3/0)
Creation of new cases too difficult	3 (1/2)
Knowledge database needed	1 (1/0)

Across the 151 comments under the theme of “missing features or needs improvement (hardware, software)”, a total of 173 thoughts were given on what impedes the use of VR-haptics in preclinical and clinical dental education.

### 3.5 Areas for improvement

When asked about functions the respondents would like to see added to VR-haptic dental trainers, 182 comments were received with a total of 217 different suggestions made. The suggestions were categorized into seven themes (Table 3). The most common suggestions were for adding in more types of training scenarios, improving the software and hardware aspects of the dental trainers, and incorporating AI-based personalization and assessment methods.

**Table 3:** Themes for improving the usability of VR-haptics in dental education. A total of 217 suggestions were made across 182 comments.

Improvement suggestion theme (*n* of mentions)	Examples
Additional training scenarios (43)	Extraction, implantology, oral surgery, injections, anesthesia, periodontics, dental fillings, identification of caries lesions, flap design, tooth transplantation, root tip resection, calculus removal, etc.
Software improvements (42)	More personalization options, voice feedback, voice controls, improved simulation of tissues, addition of surrounding structures, improved assessment options, easier upload and use of patient scans, better group-level result viewing options, etc.
Hardware improvements (42)	Portable devices, high and low-speed options for instrumentation, additional and more varied instrumentation for different training scenarios and treatments (lights, different grit sizes, etc.), cameras for tracking user posture, VR-headset use availability (in addition to 3D monitors), etc.
AI-based improvements (40)	Personalized training for students based on their strengths and weaknesses, automated performance assessment on both individual and group levels, automated tutoring, etc.
Clinical metrics additions (26)	Better real-time monitoring and analysis options, addition of (more) metrics related to tooth preparation (prosthodontics, orthodontics, crown, and abutment teeth preparation, cavity analysis), etc.
Gamification (21)	Allow users to “play” against each other for high scores (speed, precision, etc.) both locally and globally over the internet, add cooperation possibility for simultaneous work on the same case, etc.
Improved knowledge access (3)	Knowledge database/hub creation, video illustrations with subtitles

## 4 Discussion

The implementation of VR-haptic educational methods relies on suitable tools and educators' positive attitudes towards digital technologies (32). Our global survey covering 156 institutions revealed strong worldwide interest in integrating VR-haptics into dental education. This underscores the importance of identifying the potential, shortcomings, and future expectations in haptic simulators through public surveys.

### 4.1 Challenges in implementation

Integrating VR-haptic training in oral health education faces resistance, with the survey highlighting the key concerns and challenges associated with this modern approach. More than a third of the replies (35%) highlight the capabilities of the VR-haptic dental trainers as a hurdle in their implementation. This gap can affect the reliability of skill transfer from simulation to actual patient care. Enhancing the haptic precision and expanding procedural options could improve the acceptance and integration of VR-haptic systems into dental education.

More than a quarter of the responses (28%) highlighted economic challenges related to the acquisition, housing, and maintenance of these systems, leading to a shortage of available devices. Accordingly, many institutions struggle to invest in enough devices to meet student demand, which leads to limited hands-on time and prevents some programs from fully integrating these tools into their curricula. Addressing these financial constraints, possibly through funding support or cost-reduction innovations, could help increase access to these valuable educational resources.

Almost a quarter of the responses (24%) lamented low acceptance levels of VR-haptics among educators, faculty, and students. This hesitation can hinder the integration of VR-haptic systems in educational settings, as both faculty and students may be resistant to adopting new tools that disrupt established routines. Increasing training and demonstrating the educational benefits through research could help improve acceptance and foster a more supportive environment for VR-haptic dental training equipment.

Lastly, 13% of respondents noted that time constraints related to learning how to use the new equipment and adapting the curriculum for their addition can be prohibitive. Adapting the curriculum to include these simulators requires additional training sessions for faculty and students to become proficient with the equipment, which can be time-consuming. This adaptation process can take time away from other critical learning activities, making it difficult for programs to balance existing curriculum demands with the incorporation of new technology. Easily accessible databases, streamlined training, and phased integration strategies may help mitigate these time-related challenges, enabling smoother transition to VR-haptic learning.

These insights underscore the need for further development and refinement of VR-haptic systems to better meet educational standards and enhance the learning and assessments outcomes in dental training. They also align with findings from other, more localized studies where key obstacles included ineffective management, insufficient training, infrastructural limitations, and limited access to digital technologies (33).

### 4.2 Clinical phase utilization

Based on the responses, there is little resistance to adopting VR-haptic technology in preclinical settings, while its suitability for clinical phase utilization sees a 49% drop in our survey. Many factors diminish the perceived applicability of VR-haptic dental tools in a clinical context. For example, this reluctance towards its use may stem from the clinicians' resistance to moving away from traditional, patient-based educational methods. Another aspect to this may be that VR-haptic training is not seen as sufficiently relevant to the real-world aspect of practical dental education. The use of scans of patients’ oral structures could be a solution to this issue, but their use was seen as difficult by 56% of those who responded to the related question. Concerns on the effectiveness of such a format were likely a big part in this perceived difficulty, as 29% of the respondents mentioned reluctance of clinical instructors as the most challenging factor in implementing patient scans. On the other hand, lack of familiarity with modern technology was an even larger contributor to this hesitation, as 35% thought that technical proficiency is an issue. Improving this area could lead to better utilization of VR-haptics in the clinical phase of dental education. Lack of scanning equipment (19%) and concerns about patient rights and confidentiality (16%) also gained support as major factors in why patient scans are seen as difficult to use.

A lack of transferring VR-haptics applications from preclinical to clinical experiential learning is evident from the survey. The desire for discipline and subject matter-specific training scenarios for each program or institution also suggests a need for more broad and inclusive scientific interest groups and cross-discipline collaboration and training. The three most common disciplines mentioned, restorative dentistry, prosthodontics, and endodontics, would also form the most obvious multidisciplinary collaboration team that could naturally form and thus might improve future research and novel education protocols. Customized learning based on patient-specific and learner-specific VR-haptics for restorative dentistry, prosthodontics, and endodontics would be a powerful tool to train highly competent general dentists.

### 4.3 Gamification and VR-haptic dental training

During the analysis of the free-form comments, an intriguing suggestion emerged: 10% of respondents advocated for the integration of gamification elements and better interconnectedness in VR-haptics. This feedback highlights a growing interest among users in enhancing the training experience through gamified features. It has been shown that gamified dental education enhances interaction, communication, and collaboration while improving knowledge acquisition and comprehension (34–37). These immersive environments provide realistic experiences that boost student satisfaction and curiosity, while also enhancing experiential learning. Importantly, gamified sessions offer more personalized experiences, and they tend to provide more accessible adaptive feedback, making learning enjoyable and engaging for students. Free-time, gamified VR-haptic sessions with social media integration could also provide mechanisms for recognition, such as likes and shares. This validation has the potential for satisfying individuals' social needs for acknowledgment and appreciation, reinforcing their willingness to improve. Finally, gamified VR-haptic sessions might involve setting collective objectives that require teamwork. This action not only fosters collaboration but also cultivates a sense of unity as members work together towards common and individual goals (38–40). This could provide benefits in the students' overall mental wellbeing.

Overall, integrating gamification into dental education could enrich the learning process and create opportunities for unique teaching experiences, enabling students to learn in ways that were previously impossible. Due to its nature, gamified VR-haptics could be applied in various subjects across various educational levels, but especially during the formative preclinical phase.

### 4.4 Reliability of responses

Providing suggestions for improving teaching equipment is a nuanced topic, affected by several factors including individual attitudes towards technology, prior experiences with virtual learning, and the specific context of their dental research training. We learned during the analysis of the free-hand comments that of the 182 suggestion givers for device enhancements, 74% held a dental doctorate degree, with 61% also possessing a PhD. Such extensive research training may have made them more accustomed to technology, making them more receptive to adopting newer education methods, including VR-haptics and other digital platforms. Notably, more than half of the free-hand comments on potential improvements came from respondents engaged in educational research utilizing VR-haptics. Studies have shown that medical educators are increasingly integrating virtual learning due to its potential to enhance educational quality and accessibility (41–44). Those with a PhD are typically involved in research and may recognize the value of innovative digital educational tools that can enhance both teaching and learning. For instance, VR has been noted for its ability to bridge hands-on training with theoretical knowledge, which could appeal to those who prioritize evidence-based practices in education (21).

Considering this context, we believe that our data provides significant insights into critical areas for the advancement of VR-haptic technology within healthcare education. Among the suggestions for device enhancements, the top four categories identified were the need for more training scenarios, improvements at both software and hardware levels, and AI-based improvements, which each accounted for roughly a fifth of the suggestions. These reflect the factors identified as barriers to the implementation of VR-haptic technology in dental education (4, 27).

### 4.5 Future directions

Oral health care educators play a vital role in educating, training, and supporting students to become practice-ready professionals upon graduation (45). Dental education extends beyond merely training dentists; it aims to cultivate dedicated professionals committed to enhancing patient oral health and improving educational standards for future generations of dentists. This holistic approach is reflected in various educational frameworks that emphasize the importance of comprehensive training, including digital technology-reinforced traditional skills training, ethical considerations, and community health advocacy (46). American Dental Education Association and Association for Dental Education in Europe highlight that dental education programs are designed to produce graduates who not only possess technical skills but also demonstrate a commitment to lifelong learning and community service. This commitment is essential for addressing the evolving needs of patient populations and ensuring high standards in dental practice. The future of oral health education will be rooted in advanced digital health education systems. By integrating different digital technology-based training courses, we can significantly enhance the quality of education for oral health undergraduates, ultimately ensuring the oral health of our nations for years to come.

### 4.6 Strengths and weaknesses of the study

Conducting a global online survey on the utilization of VR-haptics in dental education, reaching 53 countries and 156 schools with 378 respondents, presents several strengths and weaknesses. Having such a wide outreach provided diverse perspectives and insights from experienced educators from many leading institutions. The convenience of an online survey helped lower the hurdle of participating and completing the survey, potentially helping to increase response rates. Given that a significant proportion (72%) of the survey responses were from practitioners who utilize VR-haptic training systems in their educational efforts, this study provides useful information for dental educators who wish to understand the current benefits and weaknesses of VR-haptic technologies. This study clearly shows that there is a large body of educators and researchers who are actively working towards the advancement of the effectiveness and benefits of these advanced training methods in dental education.

On the other hand, the response rate could still be higher to reach an even smaller margin of error. The response rates to this survey from outside Europe, Oceania, and North America were proportionally (population-wise) lower, likely stemming from a combination of lower VR-haptics availability and usage (economic issues) and our survey distribution channels not reaching them (social barriers). It should also be considered that participants who choose to respond to a voluntary survey may have different views from those who do not, leading to potential bias in the results. In addition, online surveys rarely manage to capture the depth of information that could be obtained through direct interviews or focus groups. These factors should be considered when interpreting the survey results to ensure a balanced understanding of the data collected.

### 4.7 Concluding remarks

In short, hardware and software aspects of VR-haptic dental trainers still need improvement to further push their adaptation and acceptance in dental schools. Lower costs, better dental discipline training scenario coverage, increased realism, and streamlined, student- and educator-friendly personalization and assessment options would make them more easily marketable to the portion of people who currently reject or are hesitant to embrace the technology. There is a real need for collaboration between restorative dentistry, prosthodontics, and endodontics educators and researchers. Establishment of easily accessible research-based databases on how to best implement VR-haptics in dental curricula and train the users are also needed to lighten the burden on the portion of educators who are actively trying to move forward its adaptation. Moving forward, it is also essential for future research to focus on how VR-haptics can specifically influence distinct aspects of the educational process or target variables related to student learning on the big scale. In addition, global surveys on students’ current perspectives on the use of VR-haptics in dental education could, in conjunction with educators' voices, lead towards the establishment of better guidelines for the use and incorporation of VR-haptics in dental education curricula. Cross-border studies such as this one will provide more nuanced insights and contribute to a more effective integration of technology in oral health education worldwide. By identifying the specific needs of teachers and the skills they may lack in implementing VR-haptics in their classrooms, the VR-Haptic Thinkers Consortium could potentially assist in developing targeted training programs. These programs would not only enhance the educators' skills but also increase their readiness to adopt such innovative approaches.

## Data Availability

The original contributions presented in the study are included in the article/Supplementary Material, further inquiries can be directed to the corresponding author.
