# Supplementary material for: Insights from the global education survey on the use of VR-haptics in dental education
Source: Front Dent Med. 2025 Apr 24;6:1576646. doi: 10.3389/fdmed.2025.1576646 (PMC12058498; doi:10.3389/fdmed.2025.1576646)
Supplement: Supplementary file 1 [file Datasheet1.pdf]

Supplementary Material

1 Supplementary Data

VR-Haptic Educator Survey

PURPOSE OF THE SURVEY

The purpose of this survey is to explore and understand educators' perceptions of Virtual Reality (VR) and Haptic training in preclinical courses. While single-centered studies exist on VR- and haptics-supported dental training, a cross-border multinational study with a substantial sample size has not been initiated before. There is also a noticeable gap in information regarding the acceptance, uses, and personal benefits for educators in VR- and haptics-supported dental education and training. The aim of this global cross-border project is to assess and review the current evidence regarding VR- and haptics-supported preclinical curriculums.

YOUR CONTRIBUTION MATTERS

Your input will contribute to advancing our understanding of the impact of VR-haptic training on dental education globally, specifically from the educator's perspective.

Participation in this research is completely voluntary, and you may choose to stop participating at any time. If you decide to withdraw your consent, the information collected up to that point may still be used as part of the research material.

According to EU ethical guidelines on privacy protection and data processing legislation in scientific studies, you will be assigned an ID number upon providing written consent. Subsequently, all data will be processed only as ID numbers, ensuring that linking data to your person is not possible. All information collected from participants via the surveys will be handled confidentially and in accordance with the legislation.

CONSENT

The completion of this survey will serve as your consent for the use of your replies in research.

The research is coordinated by the University of Eastern Finland (UEF). If you have any questions or concerns specific to your participation, please feel free to contact the local organizers at your home institution.

INSTITUTION

1. What is the university where you teach at?

   - "Carol Davila" University of Medicine and Pharmacy, RO
   - Griffith University, AU

- Heidelberg University Hospital, DE
- High Point University, USA
- Niigata University, JP
- Northern Ireland Medical and Dental Training Agency, UK
- Queen Mary University of London, UK
- Universidad de Concepción, CL
- Universidad de los Andes, CL
- Universidad Europea de Valencia, ES
- Universidad Peruana Cayetano Heredia, PE
- University of Ankara, TR
- University of Bergen, NO
- University of Debrecen, HU
- University of Eastern Finland, FIN
- University of Gothenburg, SE
- University of Helsinki, FIN
- University of Leeds, UK
- University of Liverpool, UK
- University of Nantes, FR
- University of New York, USA
- University of Stockholm, SE
- University of Tartu, EE
- University of Turku, FIN
- Other (specify)

DEMOGRAPHICS

2. What is your age bracket?

- 18-20
- 21-23
- 24-26
- 27-29
- 30-35
- 36-40
- 41-45
- 46-50
- 51 or older

3. What is your gender?

- Prefer not to say
- Woman
- Man
- Non-binary
- Other (specify)

4. What is your level of education?

(Check all that apply)

- Bachelors
- Masters
- Doctorate (PhD)
- DDS
- Not applicable
- Other (specify)

5. What is your proficiency in English?

- Poor

- Moderate
- Good
- Excellent
- Native

6. Do you have hobbies that improve your hand-eye coordination?

(Check all that apply)

- Playing video games
- Playing musical instruments
- Knitting, crocheting, or similar
- Working with wood, metal, or similar
- Modelling
- Painting
- Juggling or similar
- Origami
- Pottery
- Archery
- Drawing
- Cooking
- I do not have hobbies
- Other (specify)

VR-HAPTIC

7. How do you utilize a VR-haptic dental trainer during dental education?

(Check all that apply)

- As a supportive educational tool
- As an exclusive training method

- As a tool for evaluation of manual dexterity
- Not applicable

8. VR-haptic dental trainer is used as a supportive/additive educational method during?

(Check all that apply)

- Preclinical classes
- Predoctoral dental education
- Advanced dental education (specialist training, residency)
- Interprofessional education (specify below)
- Not applicable

9. If you have chosen "interprofessional education", specify which one:

10. Do you think the implementation of VR-haptics in PRECLINICAL graduate training is a challenge?

- Fully Agree
- Agree
- Neutral
- Disagree
- Fully Disagree

11. What is (are) the challenge(s) with the implementation of VR-haptics in PRECLINICAL graduate training?

12. Which are the courses during which a VR-haptic dental trainer is used as a supportive/additive PRECLINICAL educational method?

(Check all that apply)

- Endodontic
- Restorative
- Prosthodontic

- Implantology
- Periodontics
- Pediatric dentistry
- Restorative cariology
- Not applicable
- Other (specify)

13. Do you think the implementation of VR-haptics in CLINICAL graduate training is a challenge?

- Fully Agree
- Agree
- Neutral
- Disagree
- Fully Disagree

14. What is (are) the challenge(s) with the implementation of VR-haptics in CLINICAL graduate training?

15. Which are the courses during which a VR-haptic dental trainer is used as a supportive/additive CLINICAL educational method?

(Check all that apply)

- Endodontic
- Restorative
- Prosthodontic
- Implantology
- Periodontics
- Pediatric dentistry
- Restorative cariology
- Not applicable

- Other (specify)

16. Do you use a VR-haptic dental trainer for educational research?

- No
- Yes

17. In your own words, are there any functions that you would like to add to the VR-haptic dental trainers?

- No
- Yes (specify below)

18. If you have chosen "yes", specify which functions would you like to add to the VR-haptic dental trainers:

19. How long have you been utilizing a VR-haptic trainer?

- Less than six months
- 6-12 months
- 1-2 years
- 3-4 years
- 5 or more years
- Not applicable

20. What level of expertise do you feel you have with haptic simulators?

- None
- Novice/beginner
- Intermediate
- Advanced/proficient

21. To which degree do you feel you have expertise in teaching dental students with this technology?

- Low
- Acceptable

- High
- Very High

22. Do you feel you have received enough training to implement the use of the VR-haptic dental trainer to teach your students?

- Fully Agree
- Agree
- Neutral
- Disagree
- Fully Disagree

23. How much time do you generally dedicate to training yourself with a VR-haptic dental trainer as an educator?

- None
- Varies considerably
- Less than 1 hour/week
- Between 1 and 3 hours/week
- More than 3 hours/week

24. Do you feel you need more training hours with the VR-haptic dental trainer to get to know it better and therefore train your students better as well?

- Fully Agree
- Agree
- Neutral
- Disagree
- Fully Disagree

25. As a dental educator, when do you think is the best time to implement VR-haptic device use to support dental education?

- Before phantom head training in the preclinical stage
- After phantom head training in the preclinical stage

- Simultaneously with phantom head training in the preclinical stage
- During clinical training

26. Is implementing the use of patient scans for training a challenge?

- No
- Yes

27. If you have chosen "yes" for the previous question, what is the most challenging factor?

- Equipment for scanning
- Reluctance of clinical instructors
- Technical proficiency of clinical instructors
- Concerns regarding patient rights and confidentiality

--- *The part below was only available for the invited educators* ---

Taking part in the drawing for three 250€ prizes to help you join our 2025 London VR-Haptic Meetup

We are planning to hold our third VR-Haptic Thinkers Meetup on the 6th of June 2025 (date may be subject to change) in London, UK. If you have provided replies to this survey, are going to join the meetup on-site (rather than online), and wish to participate in the chance to win a 250€ prize to assist you in your stay at London, please provide your nickname below. Three winners will be randomly picked from the pool of given nicknames.

PLEASE NOTE that you must also fill in the form behind the Survey Prize Form button (accessible on our website through the link we sent you) so that we can contact you if you win! Make absolutely sure your nickname matches the one you provide here and that the nickname is sufficiently unique to reduce the likelihood of duplicate nicknames.

28. What is the nickname you wish to use for the prize drawing? Please create a sufficiently unique nickname to avoid overlap with other respondents.
